# Supplementary material for: The Impact of Gender and Age Differences and Infectious Disease Symptoms on Psychological Distress in Quarantined Asymptomatic or Mildly Ill COVID-19 Patients in Japan
Source: Int J Environ Res Public Health. 2022 Jul 26;19(15):9083. doi: 10.3390/ijerph19159083 (PMC9332173; doi:10.3390/ijerph19159083)
Supplement: Supplementary file 1 [file ijerph-19-09083-s001.zip › ijerph-1805843-supplementary.pdf]

Supplementary Material

exit criteria

1. Symptomatic cases
   a. 10 days have passed since the onset of symptoms and 72 hours have passed after the symptoms have abated.
   b. 24 hours after symptom resolution, and two negative PCR test results are obtained at least 24 hours apart.
2. Asymptomatic carriers
   a. 10 days have passed from the date of specimen collection.
   b. After 6 days have passed from the date of specimen collection, two negative PCR test results are obtained at least 24 hours apart.

PCR, Polymerase Chain Reaction
